# Quantitative Analysis, Extraction Optimization, and Biological Evaluation of *Cudrania tricuspidata* Leaf and Fruit Extracts

**DOI:** 10.3390/molecules22091489

**Published:** 2017-09-07

**Authors:** Seung-Hui Song, Sung Hwan Ki, Dae-Hun Park, Hong-Seop Moon, Chang-Dai Lee, In-Soo Yoon, Seung-Sik Cho

**Affiliations:** 1 Department of Pharmacy, College of Pharmacy, Mokpo National University, Muan-gun, Jeonnam 58554, Korea; tmdgml7898@naver.com (S.-H.S.); hbsmoon@mokpo.ac.kr (H.-S.M.); 2 Laboratory of Toxicology, College of Pharmacy, Chosun University, Dong-gu, Gwangju 61452, Korea; shki@chosun.ac.kr; 3 Department of Nursing, Dongshin University, Naju-si, Jeonnam 58245, Korea; dhj1221@hanmail.net; 4 Department of Business Administration, Mokpo National University, Muan-gun, Jeonnam 58554, Korea; leecdai@mokpo.ac.kr; 5 Department of Manufacturing Pharmacy, College of Pharmacy, Pusan National University, Geumjeong-gu, Busan 46241, Korea

**Keywords:** *C. tricuspidata* Bureau, simultaneous analysis, HPLC, xanthine oxidase, hyperuricemia

## Abstract

*Cudrania tricuspidata* Bureau (Moraceae) shows numerous pharmacological effects and has been used in traditional herbal remedies for inflammation, gastritis, tumors, and liver diseases. However, no validated analytical method for the standardization and optimization of the biological properties of *C. tricuspidata* preparations has been reported. We developed and validated a reverse-phase high-performance liquid chromatography (HPLC) method for the separation and quantification of active markers. Ethanolic extracts of *C. tricuspidata* leaves were prepared and evaluated for chemical profiles and biological activities. The 80% ethanolic extract demonstrated the greatest antioxidant activity and phenolic content, while the 100% ethanolic extract had the greatest total flavonoid content and xanthine oxidase (XO) inhibitory activity. The validated HPLC method confirmed that chlorogenic acid, rutin, and kaempferol were present in *C. tricuspidata* leaf extracts. We postulated that the antioxidant and anti-hyperuricemic/gout effects of *C. tricuspidata* extract could be attributed to these marker compounds. Our results suggested that the flavonoid-rich fraction of the leaf extract may be utilized for the treatment and prevention of hyperuricemia-related diseases, and the validated method and marker compounds could be applied for the quality control of *C. tricuspidata* preparations.

## 1. Introduction

*Cudrania tricuspidata* Bureau (Moraceae) is cultivated in South Korea and is used in traditional herbal remedies for inflammation, gastritis, tumors, and liver cell damage [1]. The usage of the leaf, fruit, and root of *C. tricuspidata* was reported in “Donguibogam”, one of the classics in Eastern medicine, which is used as an essential reference for the study of traditional medicine in many countries, including South Korea, China, and Japan. *C. tricuspidata* is available throughout the year and is widely used as a folk remedy. The roots and leaves of this perennial herb contain pharmaceutically active substances with anti-cancer activity, antioxidant activity, and blood glucose-lowering effects. The root bark has demonstrated anti-atherosclerotic [2], anti-inflammatory [3], antioxidant [4], neuroprotective [5], hepatoprotective [6], and cytotoxic activity [7]. In addition, *C. tricuspidata* fruit is reported to have neuroprotective [8], anti-inflammatory [9,10], pancreatic lipase inhibitory [11], mast cell activating [12], monoamine oxidase inhibitory [13], and anti-obesity effects [14]. Additionally, a previous study reported that *C. tricuspidata* leaves inhibited lipase activity, reduced plasma triacylglycerol levels, and delayed lipid absorption in vivo [15].

The efficacy of various extracts and purified active components prepared using *C. tricuspidata* as a medical material has been studied extensively to date. The root bark, fruit, and leaf are the most commonly investigated (in decreasing order of the number of publications). The contents of single compounds identified in the root bark are insufficient for use as markers for pharmaceutical industrialization. Moreover, preparations involving the fruit or leaf could be better for productivity than those involving the root bark, as *C. tricuspidata* is a perennial plant (Table 1).

Few studies have been conducted on the leaves and fruits of *C. tricuspidata*, and the contents of pharmacologically active ingredients were insufficient for use as marker compounds for pharmaceutical industrialization. Considerable effort has been focused on developing *C. tricuspidata* as medicinal and functional food materials, but no positive results have been achieved. To the best of our knowledge, no validated analytical method for the standardization and optimization of biological properties of *C. tricuspidata* preparations has been reported in the literature.

We investigated the biological activities of extracts and their constituents obtained from *C. tricuspidata* leaves and fruits for the development of medicinal or functional food sources. In this study, leaf and fruit components were screened for the medicinal application of *C. tricuspidata*. Extracts of *C. tricuspidata* were prepared for the assessment of chemical composition and pharmacological properties.

## 2. Results and Discussion

### 2.1. Optimization of the Chromatographic Conditions

**Figure 1:**
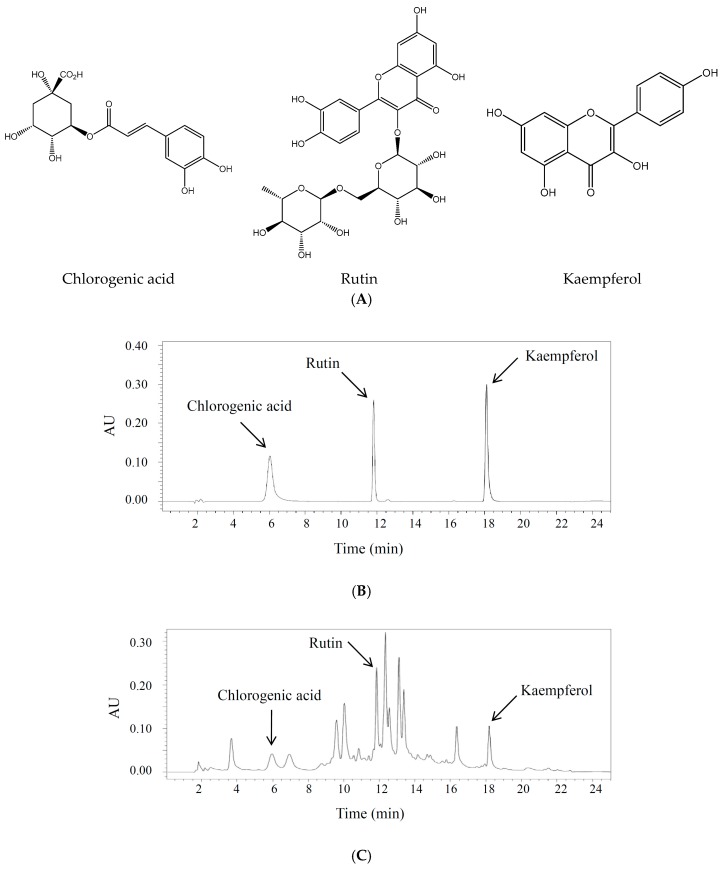

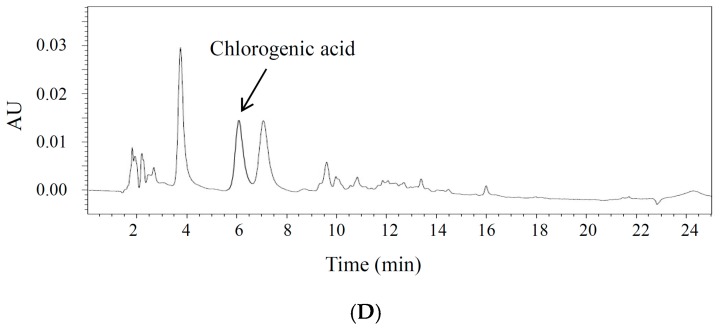
Identification of active compounds in *C. tricuspidata* leaf and fruit extracts by high-performance liquid chromatography (HPLC) analysis. (**A**) Chemical structures of chlorogenic acid, rutin, and kaempferol; (**B**) standard mixture at 340 nm; (**C**) sample extract (leaf) at 340 nm; (**D**) sample extract (fruit) at 340 nm. A mobile phase consisting of a mixture of solvent A (acetonitrile) and B (water containing 0.2% phosphoric acid) and employing a gradient elution (from 10:90 to 100:0 *v*/*v*) was run at a flow rate of 0.8 mL/min.

The HPLC conditions were optimized in terms of mobile phase composition, column temperature, wavelength, and flow rate. A gradient program was used to separate the three active markers in a single run within a reasonable period. Detection wavelengths were set according to the UV absorption maxima of the compounds (340 nm). Under the proposed analytical conditions, a baseline resolution was obtained for all analytes. The chemical structures of active constituents identified and representative chromatograms of the standard mixture and sample extracts are shown in Figure 1.

### 2.2. Method Validation

#### 2.2.1. Limit of Detection (LOD) and Limit of Quantification (LOQ)

The limit of detection (LOD) of an individual analytical procedure is the lowest amount of an analyte in a sample that can be detected but not necessarily quantified. The LOD was 0.88, 0.32 and 0.05 μg/mL for chlorogenic acid, rutin, and kaempferol, respectively (Table 1). The limit of quantification (LOQ) of an individual analytical procedure is the lowest amount of analyte in a sample that can be quantified with suitable precision and accuracy. The LOQ was 6.25 μg/mL, which is the lowest level in the linear concentration range with acceptable precision and accuracy (Table 1).

#### 2.2.2. Linearity

Calibration curves were linear over a concentration range of 6.25–100 μg/mL for the three markers. The calibration curves exhibited good linear regressions (*r*^2^ > 0.999 for chlorogenic acid, rutin, and kaempferol; Table 2).

#### 2.2.3. Precision and Accuracy

The results of the intraday and interday precision experiments are shown in Table 3. The developed method was precise, as indicated by relative standard deviation (RSD) values (<2.5%) for the repeatability of the intraday and interday precision studies, which were below the limit recommended by the International Conference on Harmonisation (ICH) guidelines [16]. The overall recovery percentages were in the range of 99.2–106.9% for chlorogenic acid, 103.6–108.3% for rutin and 103.7–107.7% for kaempferol. These results demonstrate that the developed method is reproducible with good accuracy (Table 3).

#### 2.2.4. Repeatability

The results of the repeatability experiments are shown in Table 4. The developed method was precise; the RSD values for the repeatability precision studies were below 2.0%.

### 2.3. Contents of Marker Compounds from C. tricuspidata Extracts

**Figure 2:**
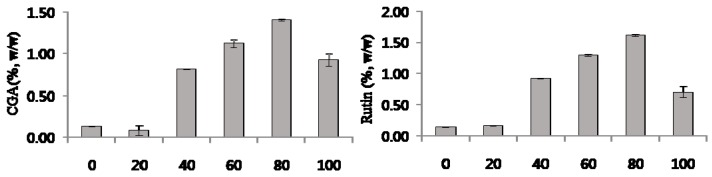
Content of chlorogenic acid (CGA) and rutin in ethanolic extracts from *C. tricuspidata* leaves; 0–100 on *x*-axis: 0–100% ethanolic extract. Each value was the mean ± SD (*n* = 3).

Plant samples were extracted with six different solvent compositions to select the best extraction solvent conditions: water, 20% ethanol, 40% ethanol, 60% ethanol, 80% ethanol, and 100% ethanol (*v*/*v*). The validated HPLC method was applied to analyze the six extracts. The average amounts (wt %) of chlorogenic acid and rutin are presented in Figure 2. The contents of the two compounds in the 80% ethanolic extract were greater than those in the other ethanolic extracts. On the basis of these results, the 80% ethanol was selected as the most effective extraction solvent.

### 2.4. Antioxidant Activity, Total Phenolic Contents, and Total Flavonoids of C. tricuspidata Extracts

The antioxidant potential of various extracts of *C. tricuspidata* was determined by the 2,2-diphenyl-1-picrylhydrazyl (DPPH) and reducing power assays. Plants are rich sources of phytochemicals with antioxidant activity [16]. Antioxidative properties of plant preparations were correlated with the major active constituents’ contents, such as polyphenols and flavonoids. Therefore, we determined the total phenolic contents and flavonoids of various extracts obtained from *C. tricuspidata*.

**Figure 3:**
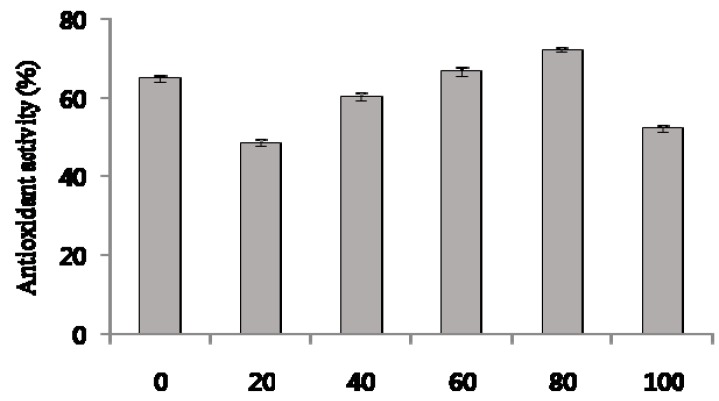
2,2-Diphenyl-1-picrylhydrazyl (DPPH) scavenging effect in ethanolic extracts from *C. tricuspidata* leaves. 0–100 on *x*-axis: 0–100% ethanolic extract. Each value was the mean ± SD (*n* = 3).

DPPH analysis is a common method for evaluating the free radical scavenging ability of plant extracts. The measured DPPH radical scavenging activity is shown in Figure 3. The antioxidant activity of DPPH radicals decreased in the following order: 80% ethanol extract (72.3 ± 0.7%) > 60% ethanol extract (66.8 ± 1.1%) > hot water extract (65.1 ± 0.9%) > 40% ethanol extract (60.1 ± 0.9%) > 100% ethanol extract (52.4 ± 0.9%) > 20% ethanol extract (48.7 ± 0.9%).

Fe^3+^ was reduced to Fe^2+^ in the presence of extracts to measure the reductive capability. The 80% ethanolic extract had the highest activity among the six extracts. The reductive activity, which was expressed as vitamin C equivalents, decreased in the following order: 80% ethanol extract (28.3 ±0.5 μg/mL) > 60% ethanol extract (27.5 ± 0.3 μg/mL) > 40% ethanol extract (26.7 ± 0.3 μg/mL) > water extract (26.3 ± 0.6 μg/mL) > 100% ethanol extract (25.8 ± 0.3 μg/mL) > 20% ethanol extract (24.9 ± 0.6 μg/mL).

The total phenolic content was determined by the Folin–Ciocalteu method [16] and was reported as gallic acid equivalents by reference to the standard curve (*r*^2^ > 0.999), as shown in Table 5. The phenolic content in the 80% ethanolic extract was higher than that in the other ethanolic extracts. The 80% ethanolic extract showed the highest DPPH radical scavenging activity, reducing power, and phenolic content.

These results suggest that solvent combinations may affect the phenolic extraction process. In our case, 80% ethanol was the most efficient solvent system for the extraction of phenolic compounds from *C. tricuspidata*. From the ethanolic extracts of *C. tricuspidata* leaves, we identified chlorogenic acid, rutin, and kaempferol. These antioxidant phenolic compounds are often found in medicinal plants and functional food sources [17,18,19].

**Figure 4:**
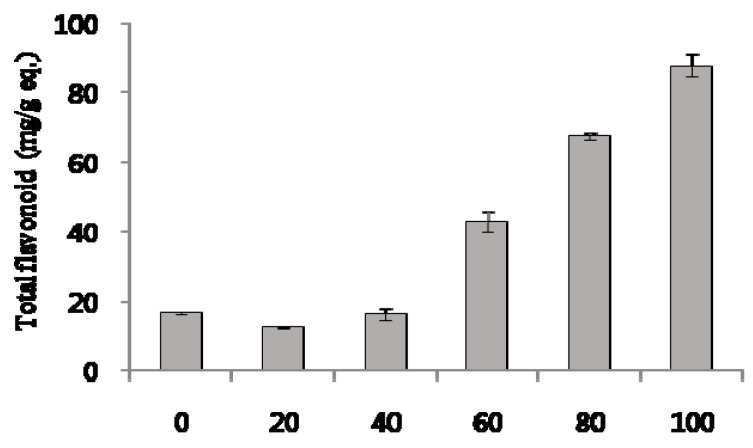
Total flavonoid content of ethanolic extract from *C. tricuspidata* leaves; 0–100 on *x*-axis: 0–100% ethanolic extract. Each value was the mean ± SD (*n* = 3).

The total flavonoid content was determined by an aluminum chloride assay [20] and was reported as quercetin equivalents by reference to a standard curve (*r*^2^ > 0.999), as shown in Figure 4. The flavonoid content in the 100% ethanolic extract was greater than that in the other ethanolic extracts. All flavonoids are phenolic compounds, but not all phenolic compounds are necessarily flavonoids. The total phenolic content in the 100% ethanolic extract was 92.8 ± 4.1 mg/g gallic acid equivalents (Table 5), and total flavonoids were 87.7 ± 3.1 mg/g (8.8% *w*/*w*) quercetin equivalents. These results indicate that the 100% ethanolic extract was the flavonoid-rich fraction. There is evidence that flavonoid-rich extracts from plant sources can exert various pharmacological effects in vitro and in vivo. Citraro et al. reported that a flavonoid-rich extract from orange juice possesses antiepileptic effects for pentylenetetrazol (PTZ)-induced seizures and in GS-sensible DBA/2 (Dilute Brown Non-Agouti) mice [21]. Huang et al. reported hypoglycemic activity and potential mechanisms of a flavonoid-rich ethyl acetate extract from *Sophora tonkinensis* Gagnep. in KK-Ay diabetic mice (type 2 diabetes) and identified 13 flavonoids [22]. However, a quantitative analysis of the flavonoid content was not performed in these previous studies. Peng et al. prepared a flavonoid-rich extract from *Maydis stigma* using a two-step extraction, and found the total flavonoid content to be 10.45% (*w*/*w*). They reported the acute toxicity, safety, and antioxidant activity of the flavonoid-rich extract in mice [23]. Gajaria et al. reported that an extract from *Murraya koenigii* alleviated in vitro low-density lipoprotein (LDL) oxidation, which induced apoptosis in murine macrophage cells [24]. In the study, leaves of *M. koenigii* were defatted overnight with 70% petroleum ether and extracted with 80% methanol. The total flavonoid content of the extract was found to be 3.1 ± 0.5 mg/g (0.3% *w*/*w*) [24]. Liu et al. reported that a flavonoid-rich extract from *Rosa laevigata* fruit shows biological effects against H_2_O_2_-induced oxidative injury in PC12 cells by adjusting oxidative stress, and suppresses apoptosis and inflammation [25]. They identified three flavonoids as key compounds in the extract, and their contents were determined to be 3.11%, 2.72%, and 1.49%, respectively [25]. The total flavonoid content of *C. tricuspidata* extracts prepared in our present study was 8.8%, which was much higher than that of *M. koenigii* as reported by Gajaria et al. [24]. and which was similar to that of *M. stigma* as reported by Peng et al. [23].

### 2.5. Xanthine Oxidase Inhibitory Activity of C. tricuspidata Extracts

**Figure 5:**
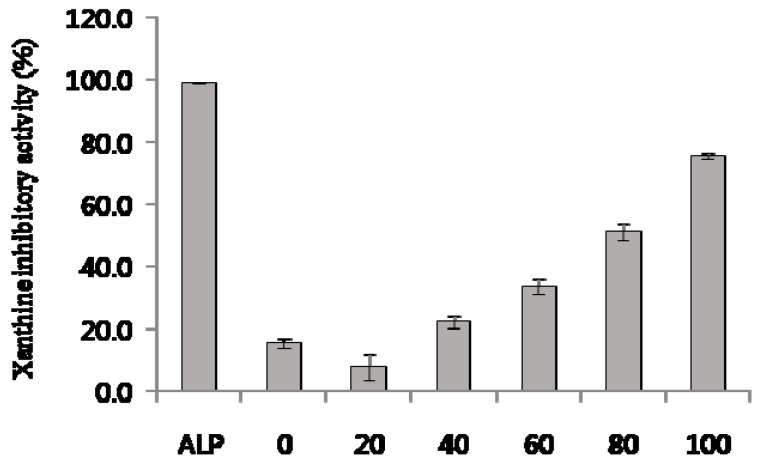
Xanthine oxidase (XO) inhibitory activites of ethanolic extract from *C. tricuspidata* leaves. ALP: allopurinol (50 μg/mL); 0–100 on *x*-axis: 0–100% ethanolic extract (2 mg/mL). Each value was the mean ± SD (*n* = 3).

Figure 5 shows the effect of ethanol concentration on the xanthine oxidase (XO) inhibitory activity of *C. tricuspidata* leaf ethanolic extracts. Figure 5 shows that allopurinol (ALP; positive control) at a concentration of 50 μg/mL significantly inhibited XO activity (98.7 ± 0.05%). The XO inhibitory activity of the ethanolic extracts significantly increased by increasing the ethanol concentration from 0 to 100% (75.4 ± 0.9%).

Previously, we had screened various medicinal sources for XO inhibitory activity. Among 229 medicinal plant extracts, 3 extracts were selected as potential XO inhibitors [26]. Extracts from *Corylopsis coreana* and *Camellia japonica* inhibited the XO at a concentration of 2 mg/mL, and relative inhibiton levels were 50% to 60% [20,27]. The extract from *Quercus acuta* inhibited the XO at a concentration of 1 mg/mL, and the inhibition level was ~50% [26]. The plant extracts with XO inhibitory activity in the range of 1–2 mg/mL demonstrated consistent effects in the in vivo animal disease model. We posit that 100% ethanolic extract of *C. tricuspidata* leaves can be developed as a candidate for anti-hyperuricemic and anti-gout medicine.

We identified chlorogenic acid, rutin, and kaempferol as marker compounds from the ethanolic extracts of *C. tricuspidata* leaves. Rutin is a flavonoid known to have various pharmacological activities, such as antioxidant, anti-convulsant, anti-inflammatory, anti-Alzheimer, and anti-hyperuricemia activities [27,28,29,30]. A previous study reported that rutin at doses of 50–100 mg/kg significantly reduced the levels of biomarkers such as serum urate, creatinine, blood urea nitrogen, and serum/kidney uromodulin in hyperuricemic mice [29]. In another study, rutin demonstrated dose-dependent hypouricemic effects by inhibiting xanthine dehydrogenase/xanthine oxidase (XDH/XO) activity [30]. Chlorogenic acid is a phenolic compound with various pharmacological effects, such as antioxidant, anti-inflammatory, anti-allergy, and anti-hyperuricemia effects [17,31,32,33,34]. Meng et al. reported that chlorogenic acid significantly reduced the Sur level by inhibiting XO activity without increasing the urinary uric acid level [32]. Chlorogenic acid also alleviates the symptoms of inflammation induced by uric acid crystals by inhibiting the production of proinflammatory cytokines, including interleukin-1β (IL-1β), interleukin-6 (IL-6), and tumor necrosis factor-α (TNF-α) [32,33,34]. These results suggest that chlorogenic acid may have considerable potential for development as an anti-gouty arthritis agent. Kaempferol is a flavonoid with antioxidant, antimicrobial, anti-inflammatory, anti-ulcer and XO inhibitory activities [18,35,36,37]. Nagao et al. reported that kaempferol inhibited XO activity at low concentrations in a mixed-type mode, suggesting that certain flavonoids might suppress the formation of active oxygen species and urate by XO in vivo [37]. As shown in Table 1, a previous study reports the anti-hyperglycemic effects of a water extract of *C. tricuspidata* leaves [15]. However, to the best of our knowledge, our present study is the first report on the process optimization of ethanolic extraction of phenolic phytochemical markers from *C. tricuspidata* leaves and the biological evaluation of the extracts and markers in terms of antioxidant and XO inhibitory activities.

Standard chemical profiling is crucial for optimization and quality control in natural source utilization. Chemical and chromatographic techniques may be used to characterize plant extracts. However, there has been no report on the standard profile for *C. tricuspidata*. Moreover, there is currently no other validated HPLC method reported for the simultaneous determination of chlorogenic acid, rutin, and kaempferol in the Moraceae family. Chlorogenic acid, rutin, and kaempferol are appropriate marker compound selections for the standardization of *C. tricuspidata* preparation and its anti-hyperuricemic/gout effects, on the basis of our results. Chlorogenic acid was also detected in the hot-water extract of *C. tricuspidata* fruit (0.18 ± 0.001%; Figure 1). Thus, our method can be used for the analysis and optimization of the *C. tricuspidata* fruit and leaf preparations.

## 3. Experimental Section

### 3.1. Plant Material and Preparation of the Extract

*C. tricuspidata* leaves and fruit were collected in May 2017 near Naju, in Jeonnam Province, Korea. A voucher specimen (MNUCSS-CT-01) was deposited in the College of Pharmacy, Mokpo National University (Muan-gun, Korea). The fruit and leaves were dried and extracted for the present study. The air-dried, powdered *C. tricuspidata* leaves and fruit (10 g) were extracted twice with 20–100% ethanol (100 mL) at room temperature for 3 days. The 0% extract was prepared using hot-water extraction (100 °C; 4 h). After filtration, the resultant ethanol solution was evaporated, freeze-dried, and stored at −50 °C. The crude extract was resuspended in ethanol and filtered using a 0.4 μm membrane. All the samples were used for extraction optimization and in vitro experiments.

### 3.2. Instrumentation and Chromatographic Conditions

All analyses were performed on an Alliance 2695 HPLC system (Waters, Millford, MA, USA) equipped with a photodiode array detector. An Agilent Zorbax extended C18 analytical column (5 μm, 150 mm × 5 mm) was used with a mobile phase consisting of a mixture of solvent A (acetonitrile) and solvent B (water containing 0.2% phosphoric acid). A gradient elution (from 10/90 to 100/0 *v*/*v*) at a flow rate of 0.8 mL/min (Table 6) was employed. The column temperature was maintained at 25 °C, and the detection wavelength was 340 nm. The solvent was filtered through a 0.22 μm filter and was degassed. The sample injection volume was 10 μL.

### 3.3. Preparation of Standards and Sample Solutions

#### 3.3.1. Standard Solutions

Accurately weighed appropriate amounts of the reference compounds (chlorogenic acid, rutin and kaempferol) were mixed and dissolved in methanol in a 100 mL volumetric flask, to obtain a stock solution of 100 μg/mL. The solutions were subsequently two-fold serially diluted to 3.125 μg/mL. The reference compounds of analytical standard grade were purchased from Sigma-Aldrich Chemical Co. (St. Louis, MO, USA).

#### 3.3.2. Sample Solutions

The crude extract (0.5 g) was dissolved in methanol (10 mL). This sample was sonicated to expedite the dissolution of particles. Subsequently, 1 mL was transferred to a volumetric flask and diluted with 9 mL of mobile phase A to obtain a final solution with a known concentration of 25 mg/mL.

### 3.4. Method Validation

The analytical method used for the quantification of chlorogenic acid, rutin and kaempferol in the ethanolic extract of *C. tricuspidata* leaves and fruit was validated in terms of specificity, linearity, sensitivity, accuracy, precision and recovery, as previously described [38,39].

#### 3.4.1. Specificity

Specificity is the ability of a method to discriminate between the study analytes and other constituents in the sample. The specificity of the HPLC method was demonstrated by the separation of analytes from other potential constituents, such as impurities, degradants, or excipients [40]. The resolution between the peaks corresponding to the main constituents found in the ethanolic extract of *C. tricuspidata* was determined by the analysis of chromatograms of the standard solution and the sample solution. This resolution was calculated using Waters Empower software (version 1, Waters, Milford, MA, USA).

#### 3.4.2. Linearity

The linearity was analyzed using three calibration curves obtained using standard solutions at five different concentrations in the range of 6.25–100 μg/mL for chlorogenic acid, rutin, and kaempferol. The data for the peak area versus the drug concentration were treated with linear regression analysis using Excel software.

#### 3.4.3. Sensitivity

The LOD was determined from the calibration curves of the chlorogenic acid, rutin, and kaempferol standards. The LOD was calculated as the SDR × 3/S, where SDR was the standard deviation of the response and S was the slope of the calibration curve. The LOQ was set to the lowest level of the calibration curves with acceptable precision and accuracy.

#### 3.4.4. Accuracy and Precision

The accuracy and precision were evaluated using recovery assays carried out by adding known amounts of the standards to the sample, at three different levels (12.5, 25 and 50 μg/mL) of the initial concentration of the sample. Each solution was injected in triplicate within one day or three consecutive days. The intraday and interday accuracy were expressed as the observed concentration relative to the true concentration. The intraday and interday precisions were expressed as the RSD.

#### 3.4.5. Recovery

Recovery was assessed by analyzing the peak areas using six determinations at three different levels, that is, 12.5, 25 and 50 μg/mL. Variations were expressed as the percentage of the standard concentration and the RSD.

#### 3.4.6. Statistical Analysis

Statistical analysis of the data was performed with Excel software.

### 3.5. Analysis of the Extract from C. tricuspidata Leaves

The HPLC method developed herein was used to quantitatively determine the amounts of the three markers in five extracts from *C. tricuspidata* leaves and fruit.

### 3.6. DPPH Free Radical Assay

Antioxidant activities of the different extracts were determined by the DPPH radical scavenging method. DPPH radicals have an absorption maximum of 517 nm, which disappears with reduction by an antioxidant compound. Ethanolic solution (1 mL) containing 1–20 mg of extract was added to a 0.4 mM DPPH ethanolic solution (1 mL). The solution was mixed, and the reaction was allowed to proceed at room temperature in the dark for 10 min. The absorbance at 517 nm was measured with a microplate reader (Perkin Elmer, Waltham, MA, USA). The radical scavenging activity was calculated as a percentage using the following equation:

DPPH radical scavenging activity (%) = [1 − (A_sample_/A_blank_)] × 100 (1)

The DPPH free radical scavenging activities of samples were compared regarding their IC_50_ (μg/mL) values.

### 3.7. Reducing Power

The reducing power of the sample was determined according to a modified reducing power assay method. The sample (0.1 mL) was added to 0.2 M sodium phosphate buffer (0.5 mL) with 1% potassium ferricyanide (0.5 mL), and incubated at 50 °C for 20 min. Following incubation, 10% trichloroacetic acid solution (0.5 mL) was added to the reaction mixture, and it was centrifuged at 2000× *g* for 10 min. The supernatant was mixed with distilled water (0.5 mL) and a 0.1% iron (III) chloride solution (0.1 mL), and the absorbance at 700 nm of the resulting solution was measured. Reducing powers of samples were expressed as vitamin C equivalents.

### 3.8. Determination of Total Phenolic Content

The total phenolic content was determined using the Folin–Ciocalteu assay. An aqueous solution (1 mL) containing 5 mg of the freeze-dried extract or standard was mixed with 1 mL of 2% sodium carbonate solution and 1 mL of 10% Folin–Ciocalteu phenol reagent. After 10 min, the absorbance was measured at 750 nm using a microplate reader (Perkin Elmer). The measurement was compared to a calibration curve of gallic acid. The results were expressed as milligrams of gallic acid equivalents per gram of the sample.

### 3.9. Determination of Total Flavonoids

The total flavonoid content was determined by a previously reported colorimetric method. Briefly, a 0.5 mL aliquot of the sample solution was mixed with distilled water (2 mL) and subsequently with 5% NaNO_2_ solution (0.15 mL). After incubation for 5 min, a 0.15 mL aliquot of 10% AlCl_3_ solution was added to the mixture, and after 5 min, 4% NaOH solution (2 mL) was added to the mixture. Water was added to the sample to bring the final volume to 5 mL, and the mixture was thoroughly mixed and allowed to stand for 15 min. The absorbance of the resultant mixture was measured at 415 nm. Then, the total flavonoid content was calculated as quercetin (QCT) equivalents (mg QCT/g extract) by reference to a standard curve (*r*^2^ = 0.999).

### 3.10. Determination of In Vitro Xanthine Oxidase (XO) Inhibitory Activity

XO inhibitory activity was measured by monitoring uric acid formation in a XO system. The assay system consisted of 0.6 mL of phosphate buffer (100 mM; pH 7.4), 0.1 mL of the sample, 0.1 mL of XO (0.2 U/mL), and 0.2 mL of xanthine (1 mM; dissolved in 0.1 M NaOH). The reaction was initiated by adding the enzyme with or without inhibitors, and the change in absorbance after 15 min of the mixture at 290 nm was measured against a reagent blank. A 0.2 mL aliquot of 1 M HCl was used to stop the enzymatic reaction. ALP was used as a positive control.

## 4. Conclusions

In the present study, ethanolic extracts of *C. tricuspidata* leaves were successfully prepared with varying ethanol contents and evaluated for their chemical profiles and biological activities. The 80% ethanolic extract was found to have the highest antioxidant activity and phenolic content, while the 100% ethanolic extract had the highest number of total flavonoids and highest XO inhibitory activity. The validated HPLC method was developed and applied to confirm that chlorogenic acid, rutin, and kaempferol were present in *C. tricuspidata* leaf extracts. These findings led us to suggest that the observed antioxidant and anti-hyperuricemic/gout effects of *C. tricuspidata* extract were attributed, at least in part, to the marker compounds. To the best of our knowledge, this is the first report on a validated analytical method for the standardization and optimization of biological properties of *C. tricuspidata* preparations. Further investigation is warranted to confirm the in vivo pharmacological activity of *C. tricuspidata* extract and its three constituents and to assess the safe use of the plant, which could lead to its potential development as an effective antioxidant and anti-hyperuricemic/gout agent.

## Figures and Tables

**Table 1:** Medicinal activity and composition of *C. tricuspidata* Bureau.

Part	Usage	Material	Reference
Leaf	Diabetes	Water extract	[15]
Fruit	Anti-obesity	6,8-Diprenylgenistein	[14]
Fruit	Neuroprotective	Cudraisoflavones B–K, 5,7,3′,4′-tetrahydroxy-6,8-diprenylisoflavone warangalone, auriculasin, erythrinin B, gancaonin B, gancaonin A, osajin, euchrenone b8, euchrenone b9, alpinumisoflavone, 40-*O*-methyl-alpinumisoflavone, 5,7,40-trihydroxy-6,8-diprenylisoflavone, erysenegalensein E, anagyroidisoflavone A, euchrenone b10, senegalensin, eryvarin B, 5,7-dihydroxy-6-(2″-hydroxy-3″-methylbut-3″-enyl)-4′-methoxylisoflavone, 3′,5-dihydroxy-4′-methoxyl-2″,2″-dimethylpyrano(6″,5″-*h*)isoflavone, derrone, 4′-*O*-methyl-200-hydroxydihydroalpinumisoflavone, isoerysenegalensein E, biochanin A, lupiwighteone, 5,3′,4′-trihydroxy-6″,6″-dimethylpyrano-(2″,3″,7,6)isoflavone, erythrinin C, and flemiphilippinin G	[8]
Fruit	Lipase inhibition	Genistein, orobol, 7,4′-dimethoxy-5-hydroxyisoflavone, genistin, oroboside 3′-*O*-methylorobol-7-glucoside, sphaerobioside, wighteone, gancaonin A 4′,5,7-trihydroxy isoflavonone, 5,7,3′,4′-tetrahydroxy-6-8-diprenylisoflavone, alpinumisoflavone, 4′-*O*-methylalpinumisoflavone, 5,3′,4′-trihydroxy-6′′,6′′-dimethylpyrano-(2′′,3′′;7,6)isoflavone, Scandenone, derrone, derrone-4′-*O*-methylether, isochandalone, ulexin B, ulexone B, (+)-dihydrokaempferol, (+)-taxifolin, (*2R*,*3R*)-7-(β-glucopyranosyloxy)-2,3-dihydro-3,5-dihydroxy-2-(4-hydroxyphenyl)-4*H*-1-benzopyran-4-one, astragalin, hirsutrin, populnin, nicotiflorin, rutin	[11]
Fruit	Anti-allergy	5,7,3′,4′-Tetrahydroxy-6,8-diprenylisoflavone	[12]
Fruit	Dermatitis	Water extract (rutin)	[12]
Fruit	Monoamine oxidase inhibition	Gancaonin A, 4′-*O*-methylalpinumisoflavone, alpinumisoflavone	[13]
Root bark	Anti-athersclerotic	Catecholic xanthones	[2]
Root bark	Anti-inflammatory	Cudratricusxanthone A	[3]
Root bark	Antioxidant	Cudranian 1, cudranian 2, kaempferol-7-*O-*β-d-glucopyranoside, quercetin-7-*O-*β-d-glucopyranoside, aromadendrin	[4]
Root bark	Neuroprotective	5,7-Dihydroxychromone, demethylsuberosin, 3-*O*-methylcudraxanthone G	[5]
Root bark	Hepatoprotective	Cudratricusxanthone A, cudraxanthone L, cudratricusxanthone E, macluraxanthone B	[6]
Root bark	Antioxidant Anti-cancer	1,3,7-Trihydroxy-4-(1,1-dimethyl-2-propenyl)-5,6-(2,2-dimethylchromeno)-xanthone catecholic xanthones	[7]

**Table 2:** High-performance liquid chromatography (HPLC) data for the calibration graphs and limit of quantification (LOQ) of the three active compounds.

Analyte	Retention Time (min)	*R*^2^	Linear Range (μg/mL)	LOQ (μg/mL)	LOD (μg/mL)
Chlorogenic acid	6.0	0.9999	6.25–100	6.25	0.88
Rutin	11.8	0.9999	6.25–100	6.25	0.32
Kaempferol	18.1	0.9999	6.25–100	6.25	0.05

**Table 3:** Analytical results of intraday and interday precision and accuracy.

Analyte	Conc. (μg/mL)	Intraday (*n* = 3)		Interday (*n* = 3)	
		RSD (%) ^a^	Accuracy (%)	RSD (%)	Accuracy (%)
Chlorogenic acid	12.5	1.7	105.6	6.1	102.6
	25	3.4	106.9	4.0	99.2
	50	0.3	106.2	4.1	102.5
Rutin	12.5	0.5	106.6	2.8	108.3
	25	1.3	103.6	2.3	105.3
	50	0.2	107.1	1.0	107.0
Kaempferol	12.5	2.8	107.6	3.0	107.7
	25	2.5	103.7	2.4	104.3
	50	1.0	107.2	1.0	106.0

**^a^** RSD: relative standard deviation.

**Table 4:** Analytical data of recovery (*n* = 6).

Analyte	Added (μg/mL)	Recovery (%; mean ± SD)	RSD (%) ^a^
Chlorogenic acid	12.5	103.6 ± 1.7	1.7
	25	101.0 ± 1.6	1.6
	50	101.4 ± 1.4	1.4
Rutin	12.5	104.0 ± 1.6	1.5
	25	99.2 ± 0.1	0.1
	50	101.4 ± 0.6	0.6
Kaempferol	12.5	104.4 ± 1.2	1.2
	25	99.2 ± 0.2	0.2
	50	101.3 ± 0.2	0.2

**^a^** RSD: relative standard deviation.

**Table 5:** Antioxidant activity and total phenolic content of *C. tricuspidata* leaf extracts.

Extract	Reducing Power (Ascorbic Acid Eq. μg/100 μg Extract)	Total Phenolic Content (Gallic Acid Eq. mg/g)
Water	26.3 ± 0.6	85.6 ± 0.9
20% EtOH Ex	24.9 ± 0.6	81.3 ± 1.4
40% EtOH Ex	26.7 ± 0.3	86.9 ± 2.1
60% EtOH Ex	27.5 ± 0.3	93.2 ± 2.4
80% EtOH Ex	28.3 ± 0.5	99.2 ± 1.2
100% EtOH Ex	25.8 ± 0.3	92.8 ± 4.1

**Table 6:** Analytical conditions of high-performance liquid chromatography (HPLC) for analysis of the three standards.

Parameter	Condition		
Column	Zorbax extended C18 (C18; 4.6 mm × 150 mm, 5 μm)		
Flow rate	0.8 mL/min		
Injection volume	10 μL		
UV detection	340 nm		
Run time	24 min		
Gradient	Time (min)	% A ^1^	% B ^2^
	0	10	90
	5	10	90
	18	50	50
	20	100	0
	21	10	90
	25	10	90

^1^ Acetonitrile; ^2^ 0.2% phosphoric acid.
